# The Next Era of Assessment: Building a Trustworthy Assessment System

**DOI:** 10.5334/pme.1110

**Published:** 2024-01-22

**Authors:** Holly A. Caretta-Weyer, Alina Smirnova, Michael A. Barone, Jason R. Frank, Tina Hernandez-Boussard, Dana Levinson, Kiki M. J. M. H. Lombarts, Kimberly D. Lomis, Abigail Martini, Daniel J. Schumacher, David A. Turner, Abigail Schuh

**Affiliations:** 1 Department of Emergency Medicine, Stanford University School of Medicine, Palo Alto, California, USA; 2 Department of Family Medicine, University of Calgary, Calgary, Alberta, Canada; 3 Kern Institute for the Transformation of Medical Education, Medical College of Wisconsin, Milwaukee, Wisconsin, USA; 4 NBME, Philadelphia, Pennsylvania, USA; 5 Johns Hopkins University School of Medicine, Baltimore, Maryland, USA; 6 Department of Emergency Medicine, University of Ottawa, Ottawa, Ontario, CA; 7 Stanford University School of Medicine, Palo Alto, California, USA; 8 Josiah Macy Jr Foundation, Philadelphia, Pennsylvania, USA; 9 Department of Medical Psychology, Amsterdam University Medical Centers, University of Amsterdam, NL; 10 Amsterdam Public Health research institute, Amsterdam, NL; 11 Undergraduate Medical Education Innovations, American Medical Association, Chicago, Illinois, USA; 12 Cincinnati Children’s Hospital Medical Center, Cincinnati, Ohio, USA; 13 Division of Emergency Medicine, Cincinnati Children’s Hospital Medical Center/University of Cincinnati College of Medicine, Cincinnati, Ohio, USA; 14 American Board of Pediatrics, Chapel Hill, North Carolina, USA; 15 Division of Emergency Medicine, Medical College of Wisconsin, Milwaukee, Wisconsin, USA

## Abstract

Assessment in medical education has evolved through a sequence of eras each centering on distinct views and values. These eras include measurement (e.g., knowledge exams, objective structured clinical examinations), then judgments (e.g., workplace-based assessments, entrustable professional activities), and most recently systems or programmatic assessment, where over time multiple types and sources of data are collected and combined by competency committees to ensure individual learners are ready to progress to the next stage in their training. Significantly less attention has been paid to the social context of assessment, which has led to an overall erosion of trust in assessment by a variety of stakeholders including learners and frontline assessors.

To meaningfully move forward, the authors assert that the reestablishment of trust should be foundational to the next era of assessment. In our actions and interventions, it is imperative that medical education leaders address and build trust in assessment at a systems level. To that end, the authors first review tenets on the social contextualization of assessment and its linkage to trust and discuss consequences should the current state of low trust continue. The authors then posit that trusting and trustworthy relationships can exist at individual as well as organizational and systems levels. Finally, the authors propose a framework to build trust at multiple levels in a future assessment system; one that invites and supports professional and human growth and has the potential to position assessment as a fundamental component of renegotiating the social contract between medical education and the health of the public.

Assessment systems are socially constructed and contextually embedded. As such, assessment criteria reflect current mental models of competence, both explicit and implicit. Assessment methods often reflect accreditation requirements and local practices which are not necessarily best practices. Human beings engaging in assessment bring biases and variable attention and abilities to the enterprise. Assessment criteria and processes subsequently reflect contemporary criteria for competence, values, and approaches to decision making. Much of assessment’s evolution to date has focused more on the technical aspects of measurement and implementation of assessment, with less attention paid to the social context of assessment which we posit has led to an overall erosion of trust in assessment [1]. This lack of attention to assessment social context represents a failure of rapport, inattention to the contextualization of assessment, and a paucity of direct feedback for continued growth and development between all stakeholders across all levels ranging from micro to global. Multiple industries and professions use assessment to demonstrate accountability to the populations they serve, by ensuring individuals demonstrate the abilities needed to meet the needs of those populations [2,3]. As assessment in medical education has evolved over time, these factors have contributed to a degradation of trust between individuals, programs, and systems [4,5]. In order to meaningfully move forward, we must seek to reestablish trust in assessment at all levels within medical education.

Assessment in medical education has evolved through a sequence of eras each centering on distinct views and values, as described eloquently by Schuwirth and van der Vleuten [6]. These eras have included measurement (e.g., knowledge exams, objective structured clinical examinations), then judgments (e.g., workplace-based assessments, entrustable professional activities) and most recently systems, or programmatic assessment, where over time multiple types and sources of data are collected and combined by competency committees to ensure individual learners are ready to progress to the next stage in their training. Ideally, the continued evolution of assessment would progress in pursuit of public and professional accountability by building on the advantages of the previous eras, preserving what is good and addressing what needs to change.

To be accountable to the public, assessment in medical education must build and maintain the trust of both learners (in that they are being fairly assessed) and society [7,8]. Trust primarily relates to medicine’s contract with the public, i.e., that the public can trust the medical system to address their health and disease specific needs, provide inclusive and compassionate care, and be entrusted with the training of a competent workforce [9,10]. It is the extension of trust from the public, and from governments on behalf of the public, which enables medicine to exist as a self-regulating profession. A trustworthy assessment system is also important for physicians-in-training as it drives learning in medicine. An assessment system that builds and retains trust from the learners and the society is by definition accountable.

Decoupling assessment from its larger social context harms trust. The current state of assessment in medical education is characterized by a myriad of complex systems-level issues. Persistent group differences in standardized test performance and harmful bias in workplace-based assessment contribute to continued oppression of marginalized groups [11,12].This is due in large part to issues of socioeconomic disparities in resources and subsequent preparedness which are far more predominant in racially and ethnically marginalized student and trainee populations [12]. Issues of power, hierarchy and learner mistreatment additionally continue to exist in the clinical learning environment [13]. The current assessment system has contributed to the production of a workforce that is not representative of the population from the perspective of both demographics and lived experiences [14,15,16,17]. As a result, many graduates within our current training programs are not considered trustworthy to care for our family members for a host of reasons, yet they are permitted to enter unsupervised practice [18]. Lastly, health outcomes in many developed, resourced countries fail to justify the cost of healthcare, and demonstrate large disparities in which only certain sectors of the public experience positive outcomes [19]. These examples along the spectrum of education, training, and healthcare delivery provide ample justification for the current state of low trust in medicine as well as medical education assessment.

Through a lens of socio-constructivism and critical theory, considering both social context and power structures, we assert that the (re)establishment of trust should be foundational to the next era of assessment. In our actions and interventions, it is imperative that we address and build trust in assessment at a systems level – or our efforts to contextualize assessment, use data in innovative ways, and create systems of anti-oppression and justice will fail. In this paper, we first review the current state of assessment with attention paid to the social contextualization of assessment and its linkage to trust. We then discuss consequences that may come to pass should the current state of low trust carry over into the next era of assessment. We posit that trusting and trustworthy relationships can exist at individual as well as organizational and systems levels. Elements of individual and systems-level trust may differ, but core elements such as integrity, reliability and competence underlie both [20]. Finally, we propose a framework to build trust at multiple levels in a future assessment system, one that invites and supports professional and human growth and has the potential to position assessment as a fundamental component of renegotiating the social contract between medical education and the health of the public.

## The Current State of Assessment

As discussed previously, ideally our current assessment efforts should keep what is good from previous eras of assessment and discard or de-emphasize what is less valuable. We believe the current era of programmatic assessment (the best “picture” of performance is formed by considering multiple sources and types of data) and the assessment as judgment era (valuing qualitative assessment data and collective decision-making regarding whether performance is on track) that preceded it offer much good [21]. These approaches represent a move beyond a sole focus on the assessment of medical knowledge into the assessment of meaningful skills and behaviors and have the ability to capture competencies as they manifest in practice, in holistic or synthetic ways [22].

In our experiences, many programs are struggling to perfect assessment approaches introduced in prior assessment eras. For example, many programs are actively learning and sharing best practices for assimilating narrative data with ratings and optimizing clinical competency committee processes and structures [23,24,25,26,27]. However, they are doing this in an environment where national leaders and regulatory bodies continue to place emphasis on psychometrics introduced in the first era of assessment. This desire to tether the field to the thinking of past eras hides a reality that is laid bare for us – traditional ways of thinking about validity and making arguments for the veracity of our validity evidence that will be convincing for key stakeholders must change. This should lead us to more thoroughly consider argumentation in light of validity, which includes a greater emphasis on the consequences of our assessments [28].

Striving for equity and fairness in medical assessments demands a refined approach that emphasizes the optimization of narrative comments. However, equity and fairness in assessment is hindered by a narrow adherence to traditional validity concepts. Ensuring assessments effectively fit their intended purpose is key to both optimizing learning and making defensible decisions regarding progression [29]. Traditional psychometric approaches rely on standardized tests and quantitative measures to assess individuals’ traits, often emphasizing numerical scores. In contrast, newer psychometric approaches explore qualitative contextual aspects of assessment, incorporating narrative data and dynamic techniques to provide a more comprehensive understanding of human behavior [30]. Evidence suggests that traditional psychometrics may inadvertently conceal biases, which is a significant concern in the context of medical evaluations. Additionally, narrative comments are increasingly cited as more useful than numerical ratings, as numerical performance is difficult to conceptualize or ascribe meaning, and these approaches often promote normative rankings and achievement orientation among learners [31,32,33]. The emphasis on these approaches promoting normative rankings and an achievement-oriented mindset among medical learners underscores the need to address these nuances. This contributes to a more comprehensive understanding of the challenges and potential improvements in achieving equity through medical assessments [34,35].

Too often, assessment is reduced to a box-ticking exercise that offers little value for learners *by design* [36,37,38]. Learners are told that the assessment system is serving them well if they simply collect a specified number of assessments. Learners even talk about “needing to get 5 more EPAs done,” turning an approach designed for considering their work (EPAs) into a tool someone handed them that they have no clear use for [36,38]. When learners view assessment in this manner, they tend to become frustrated with the assessment system within which they operate. If a program does not communicate the value of completing assessments, this then leads to their supervisors not understanding the value in assessments, and they as learners then do not understand the value in assessments. To all of these people working in the system, assessment is not about giving and receiving feedback for development, and it is certainly not about aiming that development at ensuring they are ready to meet the needs of patients. By implementing an assessment system based on a transformative idea like programmatic assessment in a superficial and transactional way, this has unintended negative consequences – this erodes trust in the system [39].

Exacerbating this situation, assessment efforts are too often a mechanism that can create distrust when assessment systems are not rigorously checked for fairness and equity and allow unwanted and harmful biases to run amuck [40]. When assessment does not strive to be fair and equitable, learners are harmed and trust is further eroded. This has become clear as biases in clerkship grades and test scores are identified, which create further issues within the leaky pipeline in medical education. As such, ensuring that assessment systems are designed with the intent to optimize equity while mitigating bias is key to ensuring a future that allows for the establishment of trust by all stakeholders in our assessments. The following sections analyze the barriers to building trust in assessment and propose a way forward to rebuild this trust in the next era of assessment.

## Barriers to Trust in Assessment

What then is standing in our way when attempting to establish trust in assessment? Assessment systems wield power and some generate revenues (e.g., national board and licensing exams for which registration fees are charged). Acknowledging such drivers is critical to the conversation of trust. We can consider the system at micro (individual learner-assessor interactions), meso (an educational program), macro (an educational institution and affiliated health system environments), and global (regulatory bodies) levels.

At the micro level, the behaviors of individuals are driven by the incentives in and hierarchy of the larger system. Both learners and assessors are conscious of the implications of assessments. In many systems, learners incur significant financial and opportunity costs to pursue medical education. Given that small differences in assessment outcomes can have significant downstream impacts on opportunity and income [41], the assessment system can foster perverse incentives to hide developmental needs rather than foster growth and the development of competency. A consumeristic structure stokes learner frustration with seemingly haphazard clinical placements coupled with an apparent lack of support for assessors (insufficient resources, training and time for direct observation and feedback) [42]. Sensitive to an apparent lack of construct validity [43,44], learners may feel driven to “gaming” the system and assessors may empathize [45].

At the meso level, educational programs are under performance pressures. Evaluation and ranking of educational programs – often via metrics that lack alignment with educational outcomes (e.g. US News and World Report rankings of medical schools) – impact funds flow and talent recruitment [46,47]. Programs are often under-resourced, leading to structures built around convenience of managing cohorts rather than tailoring to needs of individuals. This can promote an inclination among programs to defend against potential “weak” performers who may require more support. Additionally, litigation challenges to negative assessment decisions, such as probation or dismissal, create a disincentive to address competency concerns. Lacking structures and support for remediation, programs may develop an unspoken reliance on future elements or phases of the assessment system (e.g. certification examinations instead of rigorous programmatic assessment) to address serious issues, undermining the process of a self-regulating profession [48].

At the macro level, there is frequently a lack of alignment of incentives along the training program continuum (undergraduate medical education (UME), graduate medical education (GME) and continuing professional development (CPD)) and the health systems in which clinical training occurs. For example, health systems driven by throughput and margins may develop a short-term focus on efficiency at the expense of the long term benefits of the education mission. It is incumbent upon the medical education community to describe the value proposition of strengthening the integration of learning and work such that it is clear how these integrate and complement one another. Communicating the expected benefits to patient outcomes and clinician well-being that could be realized by deliberately developmental organizations may facilitate alignment [49].

At the global level, accreditors and licensing bodies developed under a historic deficit orientation to assessment and assumed an exclusionary stance. This means that accreditors and licensing bodies focused on ensuring that they theoretically excluded those who are not competent to care for patients from becoming board certified and/or licensed. Accreditors remain heavily focused on assessing consistency of process, despite the premise of competency-based medical education (CBME) demonstrating that process should vary to support desired outcomes. A lack of a shared mental model of competency across the continuum makes it challenging for licensing bodies to support local solutions while simultaneously fulfilling their function of external audit and accountability. Although steps have been taken to redesign processes to align ongoing certification with desired educational and performance outcomes, licensing and accreditation remain perceived by many as burdensome, costly, and often irrelevant to real world needs [50,51,52].

## Restoring Trust in Assessment

As we hope to move medical education into a new era of assessment, building a trustworthy system is imperative to success. Given that assessment data may be used across the continuum of medical education, we need to focus on building trust throughout all levels of this system [53,54,55,56]. We propose an assessment paradigm that is focused on trust at four levels: trust in the individual providing the assessment data (microsystem), trust in program or school leadership (mesosystem), trust in the institution and health system (macrosystem), and trust in the accrediting and licensing bodies (global assessment system). Using the framework developed by Robert Hurley to design trustworthy organizations, Table 1 provides several examples of each [20].

**Table 1:** Examples of trust-promoting interactions between learners and the medical education system across levels.


	MICRO (INDIVIDUAL)	MESO (PROGRAM)	MACRO (SYSTEM)	GLOBAL (ACCREDITING AND LICENSING BODIES)

Common Values	– Supervisor and learner dyad both value patient autonomy, are committed to patient safety, and thus prioritize shared decision making in clinical encounters	– Program is transparent in selection, advancement, and graduation processes; shares their mission; works to ensure equity in assessment and selection and is transparent in these efforts, and is committed to following these principles	– Learners aligned with institution’s mission and institution consistently delivers on its commitments to learners	– Learner and certification and licensing bodies have a shared mental model of outcomes that define a competent physician

Aligned Interests	– Supervisor and learner dyad share interest in learner growth and improvement – Learners and supervisors coproduce assessments and subsequent feedback discussions and learning opportunities to target individual needs	– Program seeks feedback from learners, makes changes with learners’ best interests in mind – Workflows and supervisor financial incentives support effort devoted to assessment and associated feedback	– Institution is clear in their commitment to education, balancing service demands on learners by employing advanced practice providers and other staff – Strategies in place to minimize tuition and learner debt	– Certification and licensing bodies and learners share interest in ensuring the provision of safe, effective, and high-quality patient care and share a vision of the relevant patient outcomes across the educational continuum – Developmental organizations recognize the business case for financial investment in education at all levels of continuum

Benevolence	– Supervisor frames assessment and the feedback that follows in context of a growth mindset, does not dwell unduly on negative outcomes	– Program community supports learners who are going through challenges	– Institutions treat learners as colleagues, including them in employee appreciation events, bonus distribution, decision-making, etc.	– Certification and licensing bodies make accommodations when learners have extenuating circumstances (e.g., appropriately consider test accommodations, extensions, and deferments) and also ensure accommodations do not need to be requested if they are consistent with common situations (e.g., time and space for pumping for breastfeeding mothers)

Competence / Capability	– Learner and supervisors have a shared mental model of competence, resulting in learners respecting supervisors’ clinical knowledge, experience, and observations which lead to the assessment and subsequent feedback – Supervisors normalize the developmental process and make sure learners know it is acceptable to not know something yet, sharing their previous and ongoing learning efforts whenever possible	– Learners expect program leadership to make necessary changes with their best interests in mind – Program provides opportunity for graduated responsibility and progressive autonomy	– GME and hospital leadership conduct themselves in a manner that allows learners to have confidence in them – Institutional leaders use learner centered data as part of continuous quality improvement (CQI) process	– Licensing and certifying bodies have activities and processes that accurately assess learners’ knowledge base and relevant outcomes – Certifying and licensing assessments are designed and rigorously tested in a multitude of contexts and with a diverse group of learners to minimize bias

Predictability & Integrity	– Supervisor provides necessary individualized support to all learners	– Program handles resident matters with confidentiality and discretion – Concerns raised by leadership or learner are handled promptly and professionally – Programmatic assessment is implemented equitably across all learners	– Institution or GME office is consistent with treatment of learners across training programs (e.g., duty hour violations or professionalism lapses are handled consistently for surgery and medical residents) – Outcomes for all learners are clearly defined and transparent in how they are assessed	– Regulatory policies are clearly written and openly shared on website so that assessed outcomes and standards are transparent and accountable to learners and the public

Communication	– There is open dialogue between learner and supervisor within feedback conversations – Supervisor maintains and encourages open communication with the learner through multiple avenues (page, email, phone number) – Supervisor is trained in providing feedback and engages in reflective practice to identify and mitigate own blind spots	– Program leadership provides regular forum to communicate with residents outside of email and phone (e.g., debriefs, feedback sessions at retreats, anonymous concern reporting)	– Institutional leaders are accessible to learners (e.g., they hold listening sessions or open houses to get to know learners)	– Learners have a seat at the table with licensing and regulatory bodies and have an avenue to provide frequent and rigorous feedback to facilitate continuous quality improvement of assessments that is incorporated into regulatory process


To enhance trust, we posit that the context of assessment in the next era must be deeply and skillfully embedded in relationships. We assert that the most immediately important relationship is the one between the learner and the individual providing them with feedback after an assessment (assessor). While the assessment source may be a teacher (faculty member), they may also be a peer or team member (fellow resident, nurse, patient’s family member). It is imperative that any assessment comes from a valid source; i.e., that the person providing the assessment be trusted by the learner and be considered credible [13,57]. However, the learner’s dependent position in the medical education hierarchy often complicates assessment from supervisory sources. Previous research has shown that learner perception of how supervisors used their power was a key influence on learner trust in their supervisors and subsequent engagement in assessment for learning [13,58]. Additionally, integrating reflection into supervisor behavior enhances learner trust and participation in their own learning. For example, the R2C2 Feedback Model promotes feedback after an assessment as an interaction in which learner engagement, supportive relationships, reflection, and cooperative planning are emphasized [59,60,61]. One challenge that exists in large institutions, particularly in the era of remote work where supervisors may leave campus after rounds to work on academic pursuits, is that frequent communication must now be scheduled rather than happening organically which under significant time pressures can hinder formation of close bonds between learner and teacher. Furthermore, learners are often pulled between multiple different learning environments which can make fully engaging in each clinical space difficult. Ideally, learners – with the help of a coach – would organize assessment data to reach specific learning goals (i.e., for a resident interested in a procedural-based fellowship, the coach may help them focus heavily on data related to procedural competence). Learners should also be encouraged to co-produce assessment data that is important to them [62]. Based on identified goals and values, learners should seek and document self-assessments and assessments from colleagues. This model will also aid in lowering the perceived stakes of frequent assessments and focus the learner’s attention on growth and development.

Sharing common values and aligned interests, displaying clinical competence, and having good communication skills all enhance a teacher’s ability to be viewed as valid and credible by the learner (Table 1). Properly weighing different sources of assessment FOR learning when using individual data points for aggregate assessment OF learning is important [63]. For example, a supervising physician whose assessment of a learner is based solely on one negative patient interaction that they witnessed is less likely to be viewed as trustworthy than one which was compiled from numerous direct observations over time. Transparency and consistency across assessment mediums is also critical; that is verbal feedback provided after an assessment should be consistent with written feedback to achieve maximum trust. When delivering verbal feedback and corresponding written narratives, we might recommend employing the four tenets of the R2C2 approach as a model for providing facilitated feedback after an assessment: 1. Building rapport and relationship; 2. Exploring reactions to and perceptions of the assessment; 3. Explore understanding of the content of the assessment; and 4. Coaching for performance change [59]. While supervising physicians often do not receive any formal training in providing feedback to residents, even a short supervisor training session can significantly improve the quality of their feedback [64].

As we move through the assessment system, these same factors are important at the program (mesosystem) level but may be harder to attain as the learner must now determine the program’s trustworthiness based on the collective values and competence of a larger number of faculty members. A program that is able to successfully convey its investment in the development and wellbeing of its learners can cultivate a psychologically safe learning environment in which learners can focus on growth and development. Including assessment in a program’s core values and mission statements can help clearly align interests of the learners and the program. For example, indicating that a program strives to graduate board certified physicians indicates the value that a given program places on certification exams as an assessment tool.

The program must then reliably deliver on its commitments and continuously assess its success in doing so to continue to foster trust with learners. Once a learner has established their goals within the training program and program leadership has reviewed and approved this, there must be a mutual investment in achieving those goals meaning the program and supervisors must partner with and support the learner in achieving those goals via individualized learning approaches. In this relationship, the learner is responsible for maintaining a growth min dset in which they see shortcomings as an opportunity to learn and improve [65,66,67]. Programs must provide the learner with ample opportunities for learning and must ensure equity and fairness in assessments and promotion decisions by working to mitigate bias and iteratively auditing assessments for potential biases.

At the macro level, institutions must commit to holding themselves, individual programs, and the health system which they oversee accountable to ensuring the attainment and maintenance of a trustworthy assessment system. This is imperative to the success of the overall functioning of the institution as well as to ensuring the optimal coexistence of learning and safe and effective patient care. This first requires that institutions, organizations, and health systems view themselves as key stakeholders in the educational system and commit to participating in the provision of education. Transparent coproduction of expectations between programs and institutions to build trust and alignment around educational outcomes particularly tied to learning and patient care instead of profits and market trends is a key step in that direction. Additionally, committing to and promoting psychological safety and equity in assessment at a systems level within the learning environment shows investment in common values between the system, programs, and learners [68,69]. Additionally, medical educators overseeing assessment programs should monitor trust across stakeholders and contribute to a broader research agenda to foster trust across institutions.

The training of learners who can safely and effectively care for patients is the primary aim of assessment at the macro level. As such, consistent, transparent regulatory support, check-ins, and communication that involves all stakeholders including programs and learners is key to demonstrate a commitment to that primary aim. This requires consistent quality assurance of the educational system, a visible commitment to equity and fairness in assessment, and recurrent discussions of the support structures and coaching available to help learners through their training and assessment in order to help them meet the clear and agreed upon outcomes required for progression. A system that consistently delivers on competence across all programs and demonstrates a commitment to both learners and patients is one that will garner the trust of all stakeholders under its umbrella.

Finally, at the global level, the power that regulatory bodies hold has historically led to inherent distrust by learners and program leadership [70,71,72,73,74]. The lack of direct contact with these assessors inhibits the ability to build trust through communication and personal relationships. Regulators at the global level must build trust through a dedication to integrity, equity, fairness, and predictability. However, regulatory and licensing bodies often view this process through a different lens – that of ensuring patient and societal safety [75,76]. Flipping this concept of trust at the global level to their viewpoint, we also owe it to another stakeholder, our patients, to ensure that society trusts the healthcare system’s ability to self-regulate and ensure that they are cared for by competent medical professionals. Society ultimately determines how trustworthy our assessments are by how much trust they have in the healthcare system.

To that end, we must reconsider our approach to licensing and certification through both of these lenses. This starts by regulatory bodies sharing priorities in assessment with those of learners and practicing clinicians as well as programs. Outcomes for certification must center around patients and society but also be relevant to learners and programs. Alignment between learners, programs, and regulatory bodies can be achieved by creating a transparent shared vision and understanding around the purpose of each assessment and its relationship to relevant outcomes to all stakeholders, particularly learners and patients. A frequent back and forth with programs and learners that builds a culture of mutual trust and respect will mitigate the feeling that certification and licensing is a “black box” that learners cannot trust. This will additionally require ensuring equity in assessment, which will require ensuring access to learning resources, mitigation of bias in assessment, and rigorous audits of systems to optimize equity between a variety of groups of learners at all levels. There must be meaningful and rigorous outcomes; however all learners must be supported in meeting those outcomes. Graded certification with these outcomes in mind will aid in trust, particularly with learners, while also ensuring patients’ needs are met [77]. Finally, transparent continuous quality improvement must be employed by regulatory and licensing bodies to demonstrate accountability to learners, programs, and society. This can be achieved by the coproduction and sharing of outcomes and measures by which regulatory and licensing bodies assess programs and learners, such that they are prepared to train future healthcare providers who are competent to care for patients and society respectively.

## Conclusions

The success of the next era of assessment hinges upon mutual trust from and between all levels: learners, program leaders, healthcare systems, accreditation bodies, and patients. It is imperative that we learn from current barriers to trust to develop trustworthy assessment systems that focus on common values and aligned interests, clinical competence, and honest communication. Future work to develop new assessment schema should incorporate co-production across all levels of the system to ensure equity and fairness for learners while maintaining focus on our profession’s ultimate responsibility to provide high-quality care to the patients we serve.
